# Developing a community-led SMS reporting tool for the rapid assessment of lymphatic filariasis morbidity burden: case studies from Malawi and Ghana

**DOI:** 10.1186/s12879-015-0946-4

**Published:** 2015-05-16

**Authors:** Michelle C. Stanton, Square Z. Mkwanda, Alexander Y. Debrah, Linda Batsa, Nana-Kwadwo Biritwum, Achim Hoerauf, Matthew Cliffe, Abigail Best, Andrew Molineux, Louise A. Kelly-Hope

**Affiliations:** Centre for Neglected Tropical Diseases, Department of Parasitology, Liverpool School of Tropical Medicine, Liverpool, UK; National Lymphatic Filariasis Elimination Programme, Ministry of Health, Lilongwe, Malawi; Kwame Nkrumah University of Science and Technology, Kumasi, Ghana; National Neglected Tropical Diseases Programme, Ghana Health Service, Kumasi, Ghana; University of Bonn, Bonn, Germany; Tripod Software Ltd, Lancaster, UK

**Keywords:** Lymphatic filariasis, Morbidity mapping, Lymphoedema, Hydrocoele, mHealth, Ghana, Malawi, SMS, Community health workers

## Abstract

**Background:**

Lymphoedema and hydrocoele are the two most common clinical manifestations of lymphatic filariasis (LF). In order to effectively target morbidity management strategies, more information is rapidly needed on morbidity burden across all endemic countries. The purpose of this study was to develop and test an SMS tool (MeasureSMS) which enables trained community-based health workers to report basic information on all cases they identified.

**Methods:**

The tool was trialled in Chikwawa district, Malawi and Ahanta West district, Ghana in 2014. Salaried health surveillance assistants (HSAs) identified and reported cases in Malawi whereas volunteer community health workers (CHWs) were used in Ghana. Health workers were trained in recognising lymphoedema and hydrocoeles and submitting individual case data using MeasureSMS, after which they undertook a LF morbidity survey. After the reporting period, a random sample of reported cases was visited by a physician to verify the health workers’ diagnoses. The proportion of correctly diagnosed cases *i.e.* the positive predictive value (PPV) was then calculated.

**Results:**

HSAs in Malawi successfully reported 256 unique cases by SMS from 107 communities (166 hydrocoele, 88 lymphoedema, 2 with both), resulting in an estimated adult prevalence of 17.7 per 10,000 and 33.0 per 10,000 for lymphoedema and hydrocoele respectively. In Ghana, despite being less experienced in using SMS, CHWs successfully reported 360 unique cases by SMS from 33 communities (169 hydrocoele, 185 lymphoedema, 6 with both), resulting in an estimated adult prevalence of 76.9 per 10,000 and 70.5 per 10,000 adults for lymphoedema and hydrocoele respectively. The verification exercise resulted in a PPV for lymphoedema and hydrocoele diagnosis of 90 % (n = 42, 95 % CI 76.5 – 96.9) and 92 % (n = 49, 95 % CI 79.5 – 97.4) in Malawi and 94 % (n = 34, 95 % CI 78.9 %–99.0 %) and 47 % (n = 59, 35.1 %–61.7 %) in Ghana, indicating that non-invasive methods for diagnosing hydrocoeles needed to be further emphasised.

**Conclusions:**

The study concludes that given the appropriate education and tools, community-based health workers are exceptionally well-placed to participate in quantifying LF morbidity burden, and other NTDs with observable symptoms. This concept has the potential to enable national programmes to more effectively monitor their community impact in an efficient, timely and cost-effective way.

**Electronic supplementary material:**

The online version of this article (doi:10.1186/s12879-015-0946-4) contains supplementary material, which is available to authorized users.

## Background

Globally, approximately 40 million people are affected by the clinical manifestations of lymphatic filariasis (LF), which ranges from acute attacks of filarial fever (acute dermatolymphangioadenitis or ADLAs) to chronic manifestations of lymphoedema and hydrocoele [1]. Managing morbidity and preventing disability amongst those already affected by LF is one of the main components of the Global Programme to Eliminate Lymphatic Filariasis (GPELF), and as such effective morbidity management strategies are vital for public health improvement. To this end, the GPELF recommends that all endemic countries should be collecting and reporting data on morbidity management by the end of 2014. This data should include the number of patients with lymphoedema and hydrocoele in each reporting unit (e.g. health centre), and further should be updated every six or twelve months [1].

Currently, there is no recommended method for collecting morbidity data, with GPELF suggesting that it could be collected either through dedicated surveys, during baseline surveys, when enumerating households during mass drug administration (MDA) for LF (assuming it is ongoing) or integrated into disability surveys conducted by non-governmental organisations [1]. Several endemic countries, including Malawi and Ghana, collect information on lymphoedema and hydrocoele cases during MDA itself, with community drug distributors noting the number of cases in their communities. Due to this task being secondary to that of drug distribution, and further due to the lack of follow-up of these reported cases, it is thought that although these figures provide an essential initial indication of the presence of LF-related morbidity, these figures may underestimate the true burden morbidity [2].

Community engagement has been shown in numerous circumstances to enhance health programmes, provided that community members are sufficiently incentivised [3–7]. For example, community health workers (CHWs) have become an integral part of the health system in many developing country settings [8], serving as a focal point in their communities for issues surrounding health and undertaking tasks such as surveillance, drug distribution and community education [6]. Their knowledge of the community is vital to disease control efforts, increasing trust between community members and the health system. There are also financial benefits to using the local workforce rather than employing an external team [9], with the added benefit that their engagement enhances the plausibility of the activity being integrated into the existing national health system.

Mobile phone technology has the potential to enhance community engagement through encouraging and simplifying the exchange of information between community members and health programmes. Examples of mobile health (or mHealth) can be found in a variety of applications including disease mapping/surveillance [10, 11], health education, monitoring treatment compliance [12], early warning systems [13] and medical supply chain management [14]. In particular, the combination of community-led health systems and mHealth tools has proven to be successful in improving HIV-infected patient care [15], real-time reporting of malaria cases [16], antimalarial supply monitoring [17] and supporting health worker-patient interactions [18, 19].

The purpose of this project was to develop and test a community-led LF morbidity reporting tool that enables local health workers to report information on members of their communities with lymphoedema and hydrocoele using short message services (SMS). The study objectives were twofold; assess whether community-based health workers (both volunteer workers and salaried employees of the national health system) were able to accurately identify members in their communities with lymphoedema and hydrocoele, and to pilot an SMS tool for reporting these identified LF morbidity cases.

## Methods

### Software

The SMS tool, *MeasureSMS*, was developed in collaboration with Tripod Software Ltd (www.tripodsoftware.co.uk). SMS data transfer using basic mobile phones as opposed to a smartphone application was chosen due to its lower cost, the greater availability of handsets, greater network availability for sending SMS messages, and the superior battery life of basic mobile phones. Further, it was anticipated that the health workers would be more likely to own a basic mobile phone as opposed to a smartphone, and hence would be able to be adopt an SMS reporting tool more readily. *MeasureSMS* is an Android-based application which enables all individual LF morbidity case records sent *via* SMS to a local smartphone to be validated (*i.e.* checked for formatting errors) and uploaded to a UK-based cloud server. Collated records could then be accessed *via* a web browser. As this smartphone uses a local SIM card, the cost of submitting each record is therefore that of sending a local SMS (MK12 ≈ £0.017 in Malawi; 0.05 cedis ≈ £0.01 in Ghana). No technological expertise were required to set up the system in the field other than ensuring that the smartphone had the app installed, and was able to connect to the internet either *via* a WiFi connection or the local 3G network.

### Study sites

The reporting tool was piloted in two endemic areas; Chikwawa district, Southern Region, Malawi and Ahanta West district, Western Region, Ghana, both of which have been shown to have a high burden of LF-related morbidity. [2, 20–24]. These two sites, including information on the roles of the health workers chosen to report LF morbidity data, are described below.

#### Chikwawa, Malawi

The SMS reporting tool was trialled in three health centre catchment areas of Chikwawa, namely St Montford/Mafale, Bereu and Kasinthula, with an estimated total population of 107,331 (75,081, 18,136 and 14,114 respectively). These three areas include approximately one fifth of the district’s inhabitants (Chikwawa population: 503,402). According to the 2008 census, 47.4 % of the population in Chikwawa are aged 18 or over, with close to half of these (49.1 %) being male. The district is served by three hospitals (Chikwawa District Hospital, Montford Hospital and Ngabu Rural Hospital), 12 health centres and 27 health posts. The study team visited the three health centre catchment areas in March 2014 for a three week period, during which the participating health workers were instructed to report information on all lymphoedema and hydrocoele cases in their catchment areas. The exercise was undertaken in collaboration with the National LF Programme.

Health surveillance assistants (HSAs) collected and reported the LF morbidity case data. HSAs are salaried employees of the Ministry of Health who are required to have completed secondary level education and a 10 week training course before assuming their role as HSA. They are usually residents in the area in which they work, and are generally responsible for 1–3 communities, which includes the supervision of volunteer CHWs in these communities.

#### Ahanta West, Ghana

The SMS reporting tool was trialled in 34 of the 114 communities in Ahanta West, which included approximately half of the district’s population (45,402 of the 106,215 inhabitants). According to the 2010 census, 54.7 % of the Western Region’s population are aged 18 or over, and of these close to half (49.3 %) are male. The district is served by Dix Cove District Hospital in addition to which there are 17 additional health providing facilities (7 clinics and 10 Community-based Health Planning and Services). The study team visited the selected communities in May 2014 for a three week period, during CHWs were instructed to report information on all the lymphoedema and hydrocoele cases in their own communities. This exercise was undertaken in collaboration with researchers at the Kumasi Centre for Collaborative Research (KCCR), Kwame Nkrumah University of Science and Technology (KNUST), Kumasi, Ghana and the National LF Programme.

CHWs collected and reported morbidity case data. CHWs in Ghana are volunteers who undertake their CHW duties in addition to paid employment, with these duties generally being conducted in the early morning and late afternoon. Usually there is one CHW assigned to each community, or which they are a resident. There are no educational requirements to becoming a CHW.

### Structure of the study

Both studies followed a very similar structure. Prior to the research team’s arrival in the area, community sensitisation took place during which the HSAs and CHWs involved in the study were contacted. All participating health workers were requested to bring their fully charged mobile phone to the training session, or to borrow one if they did not own one of their own. The training sessions were conducted by both researchers from the Liverpool School of Tropical Medicine (LSTM) and the country-based collaborators in a mixture of English and the local language *i.e.* Chichewa in Malawi and Twi in Ghana, using a translator when necessary. All written material was provided in English at the advice of the country-based collaborators.

#### Training sessions

**Fig. 1:**
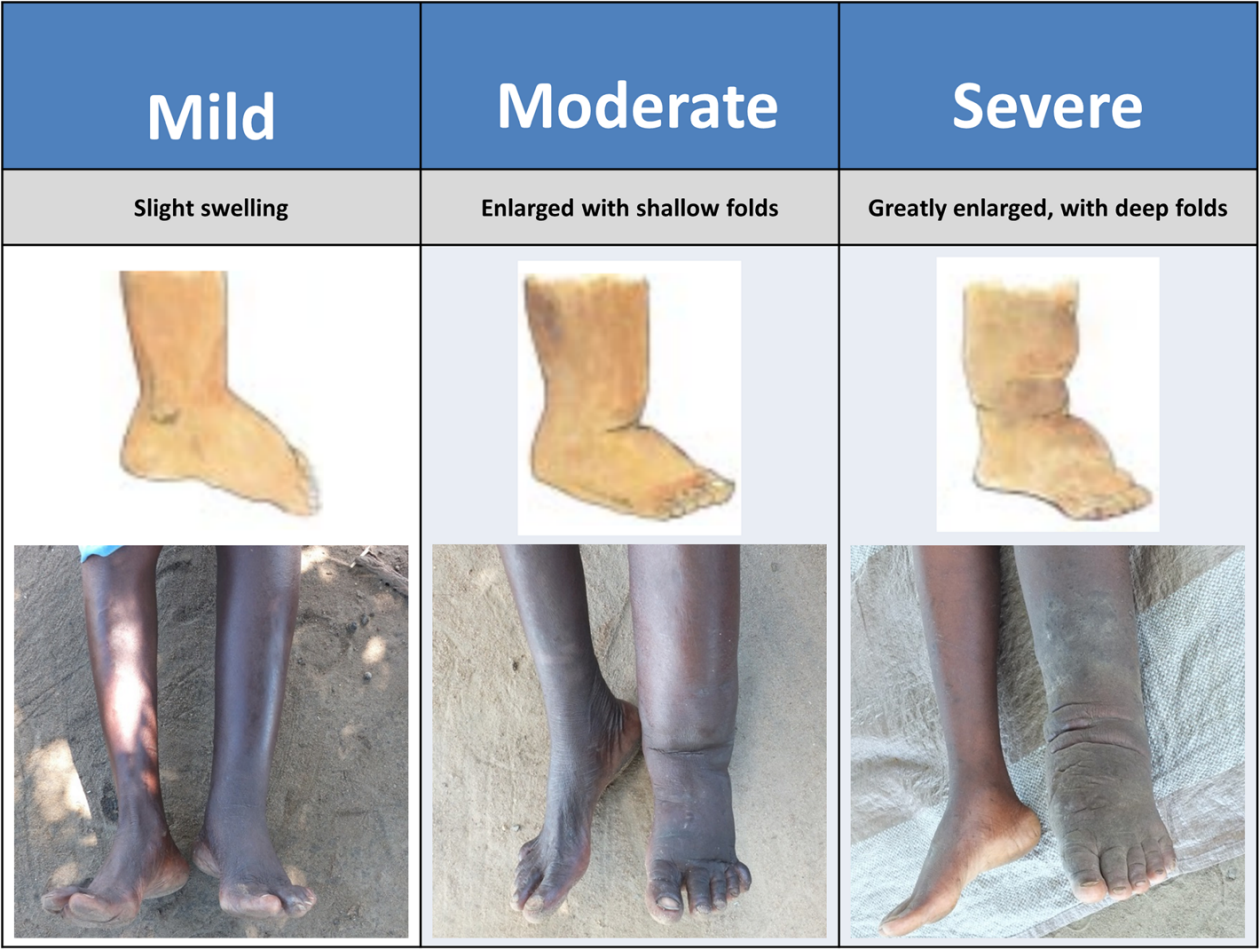
Lymphoedema severity staging used in the LF morbidity case identification study. Adapted from [27]

Full details of the structure of the training sessions, including further information on *MeasureSMS* can be found in Additional file 1. In brief, six training sessions were conducted (three per study area) during which the health workers trained in how to identify lymphoedema and hydrocoeles, how to classify lymphoedema severity into three stages *i.e.* ‘mild’, ‘moderate’ ‘severe’ (Fig. 1), [25–27], and basic lymphoedema management.

**Fig. 2:**
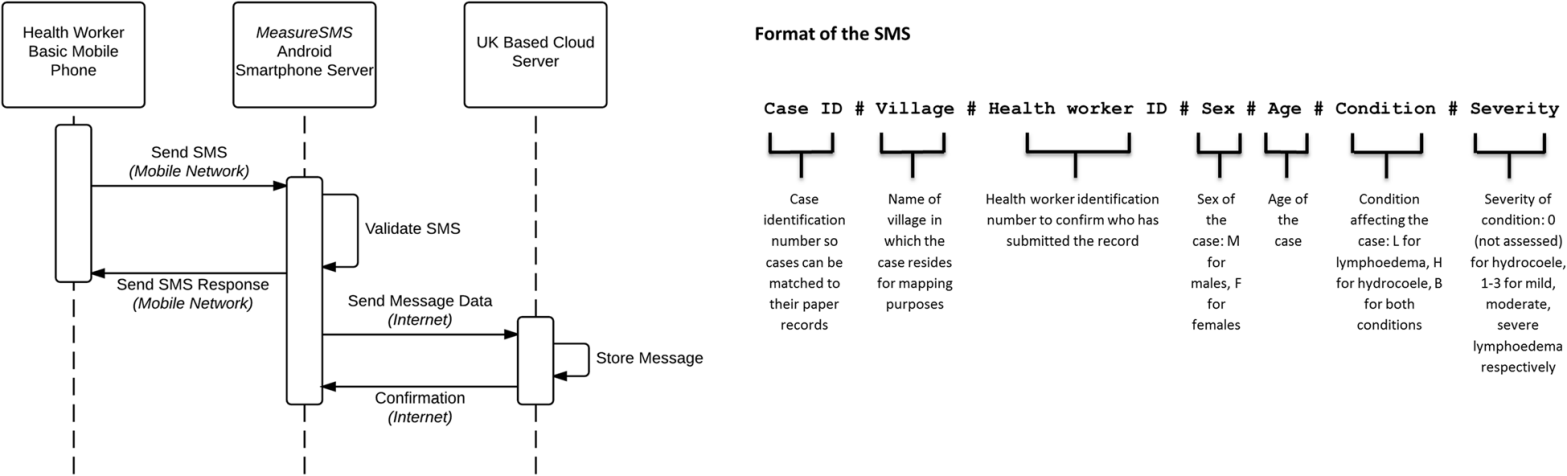
Sequence diagram MeasureSMS tool. The health worker sends their SMS record in the prescribed format to a local Android Smartphone on which the MeasureSMS app is installed. The app validates the SMS *i.e.* checks for formatting errors and sends a response SMS to the health worker indicating whether or not the SMS was correctly formatted. On connecting to the internet either *via* WiFi or the local 3G network, the smartphone sends the SMS message data to a UK-based cloud server on which it is then stored. The study team, including the members of the LF Programme can then access this stored data *via* a password protected web browser

Following this, the health workers were given further details on the data collection exercise that they were being asked to undertake. This exercise required each health worker to return to their respective catchment areas, identify all people with lymphoedema and hydrocoele within the area, record the details of each identified case on a paper form (the individual’s name, village of residence, age, sex, condition and severity of condition), then send an SMS containing this information to a smartphone on which the *MeasureSMS* app was installed. The data reporting process is presented in Fig. 2. During this period, a practise data reporting exercise was conducted. At the end of the training session, health workers were asked to complete a pre-study questionnaire to obtain demographic information and assess their initial level of confidence in identifying cases in their communities, and reporting this information using the SMS system (Additional file 2).

#### Data collection period

Each health worker was then given 4–6 days to collect information on lymphoedema and hydrocoele cases in their catchment areas. During this period if it was observed that a health worker had yet to submit any information they were contacted by telephone or visited by the research team to ensure that they were not encountering any problems. Health workers were encouraged to submit all of their data within the given timeframe, however any records submitted up to two weeks after this were included in the final dataset, but excluded from the sampling frame for the verification exercise (below).

#### Verification exercise

A subset of cases submitted by the health workers were examined by a physician to verify the diagnosis and severity (for lymphoedema only) that had been reported. This information was then used to calculate the positive predictive value (PPV) of the reported cases *i.e.* the proportion of reported cases that were true cases. Estimates of lymphoedema and hydrocoele prevalence plus health worker PPVs estimates were obtained from a study conducted by [2, 28]. Based on this it was estimated that at least 27 lymphoedema cases and 49 hydrocoele cases in Malawi, and 23 lymphoedema cases and 39 hydrocoele cases in Ghana would need to be verified in order to estimate PPV with a 10 % precision (see Additional file 3).

Stratified random sampling was used to select lymphoedema cases, where cases were stratified by severity of condition (both studies) and sex (Ghana only), where the proportion of cases in each strata was determined by the sex and severity distribution of all reported cases. No stratification was undertaken when selecting hydrocoele cases. The stratified random samples were obtained by the study team using a random number generator. All selected cases were then contacted by their health worker, and a time period was then arranged for the research team (including a physician) to visit the individuals either in their homes, or at a designated temporary clinic *i.e.* a local school or health facility. If a selected case was known to be unable to participate prior to travelling to their community, a replacement reported case was selected randomly from the list of reported cases. For logistical purposes, if on arrival to their community a selected case was unable to participate in the verification exercise, a reported case (of the same lymphoedema severity and sex if appropriate) from the same or neighbouring community was included in the verification sample where possible.

The physician examined each of the selected patients to verify their condition and its severity. Dreyer staging was used to classify lymphoedema; stages 1–2 = ‘mild’, stages 3–4 = ‘moderate’, and stages 5–7 = ‘severe’ [25, 29]. To aid hydrocoele diagnosis, the absence of a cough impulse was assessed (Malawi and Ghana), and a pen torch was used to assess for transillumination (Ghana only) [30]. All cases diagnosed with lymphoedema were provided with a basic hygiene kit (bowl, soap and towel) and were given advice on how to effectively manage their lymphoedema to prevent acute attacks, whereas all cases diagnosed with hydrocoele or inguinal hernias were given advice on how to access surgery. In both study areas hydrocoelectomies were available free of charge at the District Hospital, although cases in Ghana also required suitable health insurance.

In addition to calculating the PPV, similarities between the assessed severity of lymphoedema made by the health workers and the physician were assessed for all confirmed lymphoedema using Cohen’s Kappa coefficient [31].

#### Comparison with alternative sources

This study design does not allow the sensitivity of the data collection method to be calculated *i.e.* the proportion of all true lymphoedema cases in the study communities that were reported by the health workers. In order to obtain information on unreported cases, alternative morbidity information sources were sought. In Malawi these were limited to morbidity data collected during MDA, which has previously been shown to underestimate morbidity prevalence [2]. For Ghana, in addition to MDA reports, more comprehensive data were available in the form of an LF morbidity survey conducted by researchers at KCCR, KNUST in 2006 and 2009 [24, 32, 33].

#### Health worker feedback

Health workers were further asked to complete a post-study questionnaire to provide further information on their experiences of the data collection (Additional file 4). Health workers in Ghana were also invited to a focus group discussion to further elucidate their opinions.

### Ethical approval

Ethical approval for this study was obtained from LSTM’s Research Ethics Committee, the National Health Sciences Research Committee, Ministry of Health, Malawi and the Committee on Human Research, Publications and Ethics, KNUST, Ghana. Informed consent was obtained from all health workers involved in the study, and all lymphoedema and hydrocoele cases who were included in the verification exercise.

## Results

### Training sessions

**Fig. 3:**
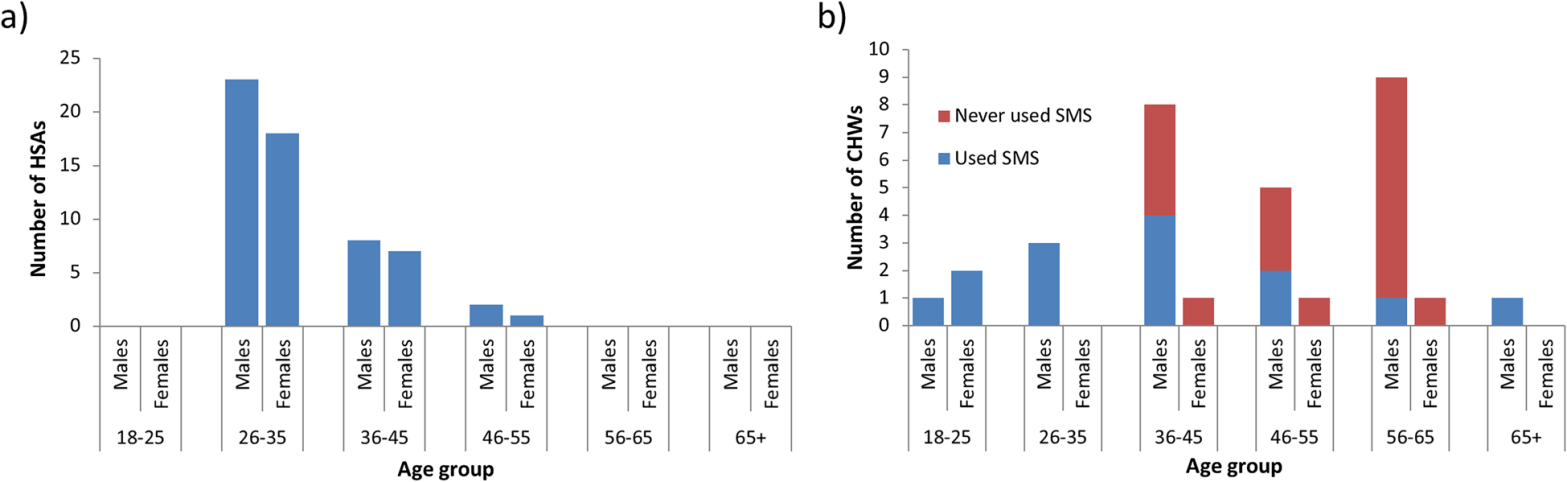
SMS experience by age and sex of participating health workers in Malawi (**a**) and Ghana (**b**)

A total of 60 HSAs covering 126 communities in Malawi and 32 CHWs covering 34 communities in Ghana participated in the training exercises. In comparing the demographics of the two groups, close to half of the HSAs in the Malawi study were female (43 %, 26/60) whereas only 16 % (5/32) of CHWs were female in Ghana. There were further differences in the age distribution of health workers, with HSAs generally younger than the CHWs. As anticipated the salaried HSAs were on average more educated than voluntary CHWs, with 85 % (51/60) completing secondary education in comparison to 19 % of CHWs (6/32) (Table 1, Fig. 3).

**Table 1:** Age and education level summaries of HSAs (Malawi) and CHWs (Ghana) trained to undertake the LF morbidity reporting exercise

	Malawi						Ghana					
	Males		Female		Total		Males		Female		Total	
Age group	N	(%)	N	(%)	N	(%)	N	(%)	N	(%)	N	(%)
18-25	0	(0 %)	0	(0 %)	0	(0 %)	1	(4 %)	2	(40 %)	3	(9 %)
26-35	23	(68 %)	18	(69 %)	41	(68 %)	3	(11 %)	0	(0 %)	3	(9 %)
36-45	8	(24 %)	7	(27 %)	15	(25 %)	8	(30 %)	1	(20 %)	9	(28 %)
46-55	2	(6 %)	1	(4 %)	3	(5 %)	5	(19 %)	1	(20 %)	6	(19 %)
>55	0	(0 %)	0	(0 %)	0	(0 %)	10	(37 %)	1	(20 %)	11	(34 %)
Missing	1	(3 %)	0	(0 %)	1	(2 %)	0	(0 %)	0	(0 %)	0	(0 %)
**Education**												
Completed Primary	0	(0 %)	2	(8 %)	2	(3 %)	6	(22 %)	2	(40 %)	8	(25 %)
Some secondary	5	(15 %)	2	(8 %)	7	(12 %)	18	(67 %)	0	(0 %)	18	(56 %)
Completed secondary	29	(85 %)	22	(85 %)	51	(85 %)	2	(7 %)	1	(20 %)	3	(9 %)
More than secondary	0	(0 %)	0	(0 %)	0	(0 %)	1	(4 %)	2	(40 %)	3	(9 %)
**Total**	34		26		60		27		5		32

Whilst mobile phone ownership was comparable between the two groups, with 93 % (56/60) of HSAs and 88 % (28/32) of CHWs owning their own phones, stark differences were observed in mobile phone use (Fig. 3). All HSAs had previously used mobile phones to send SMS messages, and expressed that they were confident in doing so. Further, all HSAs reported sending an SMS as part of their role, with 72 % of HSAs indicating that they had sent patient information using SMS in the past. In contrast, only 44 % (14/32) CHWs had prior experience of sending SMS messages, and of these only 29 % (4/14) had sent an SMS as part of their CHW duties. Confidence in sending SMS messages improved during the training session such that before training, 63 % (20/32) of CHWs stated that they had no confidence in sending SMS messages, compared to 25 % (8/32) of CHWs after training (p = 0.011). In order to address this technological barrier in Ghana, CHWs were given the option to appoint an assistant. An extra training session was arranged for these assistants to ensure they understood the objectives of the data collection exercise.

With regard to LF morbidity, following the training 98 % (59/60) of HSAs and 94 % (30/32) of CHWs were confident that they could identify lymphoedema and hydrocoele cases in their communities, although it should be noted that no distinction was made between the two conditions in this instance. Health workers also expressed a high level of confidence in assessing the severity of lymphoedema (59/60 HSAs, 31/32 CHWs), and in advising cases on how to manage their lymphoedema/hydrocoele (59/59 HSAs, 29/32 CHWs).

### Reported data

#### Malawi

In Malawi, 368 records were submitted *via* SMS over a period of 17 days. Of these, 64 (17 %) contained formatting errors and 28 (8 %) were duplicates, resulting in 276 unique records in the final dataset. One HSA included in the study covered villages that were outside of the selected catchment areas, hence the 7 records submitted by this HSA (5 hydrocoele, 2 lymphoedema) have been omitted from the remaining summaries. Further, 13 reported cases were under the age of 18. These were also excluded from the summaries as this study focused on the prevalence in the adult population only.

Of these 256 unique records, 166 people (65 %) from 107 communities were identified as having hydrocoele only, 88 people (34 %) were identified as having lymphoedema only, and 2 people (1 %) were identified as having both hydrocoele and lymphoedema (Table 2). This equated to a lymphoedema prevalence of 17.7 per 10,000 adults and a hydrocoele prevalence of 33.0 per 10,000 adults. Of those 90 people with lymphoedema, 50 people (56 %) were identified as having a mild lymphoedema, 18 (20 %) were moderate, and 18 (20 %) were severe. Four people had a severity score of zero/unclassified (3 males, 1 female), implying that they either had a breast lymphoedema, the severity could not be assessed, or they were misreported by the CHW. Further details on the number of reported cases per community, plus their respective GPS coordinates, can be found in Additional file 5.

**Table 2:** Summary of unique cases reported by HSAs (Malawi) and CHWs (Ghana) by SMS

Study area	No of hydrocoeles	No of lymphoedema					Total
		Mild	Moderate	Severe	Unknown	All	
**Malawi**							
St Montfort/Mafale	82	14	11	9	2	36	118
Bereu	47	15	1	1	1	18	65
Kasinthula	39	21	6	8	1	36	75
Total	168	50	18	18	4	90	256
**Ghana**							
Ahanta West	175	100	62	29	0	191	360

The age and sex distribution of the reported lymphoedema cases is presented in Table 3. In general, females were more likely to have lymphoedema than males (68 % [61/90] females, 95 % CI 57.0 %–77.0 %), and males were marginally younger than (adult males median = 55; adult females median = 60). No statistical association was found between sex and severity (p = 0.333) or age group and severity (p = 0.398). The age of reported hydrocoele cases ranged between 20–90, with a median of 51 years.

**Table 3:** Age and sex distribution of reported lymphoedema cases by severity and study location

		Malawi								Ghana							
		Reported Lymphoedema severity								Reported Lymphoedema severity							
		Mild		Moderate		Severe		Total		Mild		Moderate		Severe		Total	
**Sex**	**Male**	12	(24 %)	7	(39 %)	7	(39 %)	29	(32 %)	28	(28 %)	18	(29 %)	8	(28 %)	54	(28 %)
	**Female**	38	(76 %)	11	(61 %)	11	(61 %)	61	(68 %)	72	(72 %)	44	(71 %)	21	(72 %)	137	(72 %)
**Age**																	
	**18-25**	1	(2 %)	1	(6 %)	0	(0 %)	2	(2 %)	7	(7 %)	1	(2 %)	0	(0 %)	8	(4 %)
	**26-35**	6	(12 %)	1	(6 %)	3	(17 %)	10	(11 %)	13	(13 %)	4	(6 %)	1	(3 %)	18	(9 %)
	**36-45**	8	(16 %)	2	(11 %)	1	(6 %)	11	(12 %)	21	(21 %)	14	(23 %)	6	(21 %)	41	(21 %)
	**46-55**	8	(16 %)	4	(22 %)	2	(11 %)	16	(18 %)	22	(22 %)	11	(18 %)	14	(48 %)	47	(25 %)
	**56-65**	15	(30 %)	4	(22 %)	3	(17 %)	22	(24 %)	24	(24 %)	13	(21 %)	6	(21 %)	43	(23 %)
	**66-75**	7	(14 %)	4	(22 %)	5	(28 %)	17	(19 %)	8	(8 %)	11	(18 %)	2	(7 %)	21	(11 %)
	**>75**	5	(10 %)	2	(11 %)	4	(22 %)	12	(13 %)	5	(5 %)	8	(13 %)	0	(0 %)	13	(7 %)
	**Total**	50		18		18		90		100		62		29		191	

Whilst in the field it was possible to compare 58 % (35/60) HSAs’ paper records to the corresponding SMS records to determine whether any errors had been introduced whilst writing the SMS. A total of 166 cases had been reported by these HSAs *via* SMS whereas 168 records were recorded on their paper forms, with two HSAs over-reporting the number of cases, and three HSAs under-reporting. Of those reported, 7 % (11/166) misreported the age of cases and 2 % (4/166) misreported condition.

Further to the cases reported during the designated reporting period, 39 HSAs reported an additional 41 ‘missed’ cases (6 lymphoedema, 35 hydrocoele) *i.e.* cases they believed to be residing in their communities but who they were unable to meet with during the study period. Hence, the reporting rate for these 39 HSAs was 72 % (105 cases reported, 146 cases suspected). Condition specific reporting rates were 86 % (38/44) for lymphoedema and 66 % (68/103) for hydrocoele. It was not possible to obtain ‘missed’ cases information from the remaining 21 HSAs.

#### Ghana

A total of 17 CHWs and 12 assistants reported case data; two assistants were each assigned two CHWs, and further two CHWs from one village were trained, with only one CHW submitting data. Of these 12 assistants, 7 assisted in sending the SMS messages only whilst the remaining 5 assisted in case identification. Ten of these assistants attended the assistants training sessions whereas the remaining two informally assisted the CHWs during the data collection exercise.

A total of 549 records were submitted *via* SMS over a period of 21 days. Of these, 86 (16 %) contained formatting errors and 74 (13 %) were duplicates, resulting in 389 unique records in the final dataset. Of these, six cases were reported from outside of the study villages and a further 23 of the reported cases were under the age of 18, hence these cases are excluded from the subsequent summaries.

Of the 360 unique records, 169 people were identified as having hydrocoele only, 185 people were identified as having lymphoedema only, and 6 people were identified as having both hydrocoele and lymphoedema (Table 3). This equated to a lymphoedema prevalence of 76.9 per 10,000 adult population and a hydrocoele prevalence of 70.5 per 10,000 adults. Of those 191 people with lymphoedema, 100 (52 %) people were identified as having a mild lymphoedema, 62 (32 %) were moderate, and 29 (15 %) were severe. Further details on the number of reported cases per community, plus their respective GPS coordinates, can be found in Additional file 5.

The age and sex distribution of the reported lymphoedema cases is presented in Table 3. A similar sex ratio to the Malawi data was observed *i.e.* 72 % (137/191) of cases were female (95 % CI for females = 65.7 %–77.9 %), with males once again being generally younger than females (adult males median = 44; adult females median = 54). No clear trend was observed between gender and severity (p = 0.986) or age and severity (p = 0.352). The age of reported hydrocoele cases ranged between 18–95, with a median of 45 years.

Whilst in the field it was possible to compare 81 % (26/32) of CHWs’ paper records to the SMS records to determine whether any errors had been introduced whilst writing the SMS. A total of 328 cases (81 % of all records) had been reported by these CHWs *via* SMS whereas 329 records were recorded on their paper forms due to one CHW failing to report a case by SMS. Of those reported, 1 % (3/328) misreported the age of cases and three CHWs misreported severity of lymphoedema.

### Verification exercise

#### Malawi

Over a two week period, 89 identified cases (34 % of the 258 reported) from 54 villages were visited by the research team: 47 reported as having a hydrocoele only, 40 reported as having a lymphoedema only, and 2 reported as having both conditions. Of the 42 reported lymphoedema cases, 20 (48 %), 11 (26 %), 10 (24 %) were reported as having a severity assessment of mild, moderate and severe respectively. One case (2 %) was reported as having a severity of zero, implying that the individual had a breast lymphoedema. However, as this person was male, it is thought that this severity score was sent in error.

Of the 49 identified hydrocoele cases visited by the research team, 45 were confirmed as having a hydrocoele (Table 4), resulting in a positive predictive value of 0.92 (95 % CI [0.795, 0.974]). Of those four cases misdiagnosed, two were diagnosed with inguinal hernia. Further, 20 % (9/45) of those diagnosed with hydrocoele were also diagnosed with hernia, resulting in 22 % (11/49) of the 49 cases identified by the CHW having inguinal hernia and 96 % (47/49) of cases having either hydrocoele, inguinal hernia or both. For lymphoedema, 38 of the 42 reported cases were confirmed, resulting in a PPV of 0.90 (95 % CI [0.765, 0.969]). There was however substantial disagreement in the assessment of severity of those 38 with confirmed lymphoedema (Table 5) resulting in a Cohen Kappa value of 0.47 (95 % CI [0.24, 0.69]). The greatest disagreement occurred in the ‘moderate’ category such that of the 13 lymphoedemas classed by the HSAs as moderate, only half (6/13) were confirmed as being in this category by the physician, with the remaining 7 cases being split between mild (4 cases) and severe (3 cases).

**Table 4:** Summary of verification data

	Identified Lymphoedema		Identified Hydroceole		
	N	Lymphoedema PPV (95 % CI)	N	Hydrocoele PPV (95 % CI)	Hydrocoele + hernia PPV (95 % CI)
**Malawi**					
St Montfort/Mafale	23	0.87 (0.653, 0.966)	26	0.92 (0.734, 0.987)	0.96 (0.784, 0.998)
Bereu	8	0.88 (0.467, 0.993)	15	0.93 (0.660, 0.997)	1.00 (0,747, 1.00)
Kasinthula	11	1.00 (0.679, 1.000)	8	0.88 (0.467, 0.993)	0.88 (0.467, 0.993)
Overall	42	0.90 (0.765, 0.969)	49	0.92 (0.795, 0.974)	0.96 (0.849, 0.993)
**Ghana**					
Ahanta West	34	0.94 (0.789, 0.990)	59	0.47 (0.351, 0.617)	0.71 (0.571, 0.815)

**Table 5:** Assessment of the agreement in lymphoedema severity between the physician and the health worker

		Health worker assessment		
		Mild	Moderate	Severe
**Malawi**				
**Physician’s assessment**	**Mild**	12	4	0
	**Moderate**	1	6	4
	**Severe**	1	3	6
**Ghana**				
**Physician’s assessment**	**Mild**	3	2	0
	**Moderate**	10	7	0
	**Severe**	1	3	4

#### Ghana

Over a five day period, 92 identified cases (26 % of those reported) from 25 villages were visited by the research team: Of these 92 cases, 58 had been reported as having a hydrocoele only, 33 had been reported as having a lymphoedema only, and one case was reported as having both conditions. Of the 34 reported lymphoedema cases, 18 (53 %), 11 (32 %), 5 (15 %) were reported as having a severity assessment of mild, moderate and severe respectively. One selected female case was reported as having a hydrocoele.

For lymphoedema, 32 of the 34 reported cases were confirmed (Table 4), resulting in a PPV of 0.94 (95 % CI [0.789, 0.990]). There was however substantial disagreement in the assessment of severity of those 32 with confirmed lymphoedema (Table 5) resulting in a Cohen Kappa value of 0.19 (95 % CI [-0.07, 0.46]). The greatest disagreement occurred in the ‘mild’ category such that of the 14 lymphoedemas classed by the CHWs as mild, only 21 % (3/14) were confirmed as being in this category by the physician, with 10 being classed as moderate, and 1 being classed as severe.

Of the 58 identified hydrocoele cases visited by the research team, 28 were confirmed as having a hydrocoele (Table 4), resulting in a positive predictive value of 0.47 (95 % CI [0.351, 0.617]). A large proportion (28/58) of those misidentified by the CHW had inguinal hernia, and further 46 % (13/28) of verified hydrocoele cases were also diagnosed with inguinal hernia, resulting in 71 % (41/58) of the 58 cases identified by the CHW having inguinal hernia and 95 % (55/58) of cases having either hydrocoele, inguinal hernia or both. With respect to the reported female hydrocoele case, it should be noted that this was not a reporting error, but instead was an instance where the CHW suspected that the female had a hydrocoele, thus demonstrating their misinterpretation of the condition. After examination, this female was suspected of having a lipoma.

### Comparison with alternative sources

#### Malawi

Annual reported lymphoedema and hydrocoele figures collected during MDA are collated at the health centre catchment area. Within the three catchment areas, eight HSAs (7 in St Montfort/Mafale, 1 in Bereu) were unable to participate in the study, hence the figures collected during this study underestimate the burden. Using data collected during 2013, a total of 30 lymphodema (3 in St Montford/Mafale, 14 in Bereu, 13 in Kasinthula) and 63 hydrocoeles (26 in St Montford/Mafale, 31 in Bereu, 6 in Kasinthula) were reported (Tables 6 and 7). Hence, despite the missing data, using the most conservative estimates of prevalence obtained from this study *i.e.* after adjusting for PPV and ignoring the anecdotal under-reporting estimates, lymphoedema prevalence estimates are three times greater than those obtained during MDA (15.9 per 10,000 adults in comparison to 5.9 per 10,000 using MDA reports). Similarly, adjusted hydrocoele prevalence estimates are more than double those obtained during MDA (30.5 per 10,000 adults vs 12.4 per 10,000 adults).

**Table 6:** Comparison of lymphoedema data with MDA reports

Lymphoedema	Estimated Adult Population	Reported	Adjusted by PPV	Adjusted by under-reporting estimate and PPV	Reported during MDA^a^
**Malawi**					
St Montfort/Mafale	35,588	36	31	37	3
Bereu	8,596	18	16	18	14
Kasinthula	6,690	36	36	42	13
Total	50,874	90	81	94	30
**Ghana**					
Selected communities in Ahanta West	24,835	191	180	N/A	13

^a^MDA figures include children

**Table 7:** Comparison of hydrocoele data with MDA reports

Hydrocoele	Estimated Adult Population	Reported	Adjusted by PPV	Adjusted by under-reporting estimate and PPV	Reported during MDA^a^
**Malawi**					
St Montfort/Mafale	35,588	82	75	114	26
Bereu	8,596	47	44	66	31
Kasinthula	6,690	39	36	55	6
Total	50,874	168	155	234	63
**Ghana**					
Selected communities in Ahanta West	24,835	175	82	N/A	12

^a^MDA figures include children

#### Ghana

Community-level MDA data obtained from the national LF programme indicated that only 13 lymphoedema cases and 12 hydrocoele cases were reported by the communities included in this study. These figures grossly underestimate the LF morbidity prevalence in the area, with lymphoedema prevalence in adults reported during this study (adjusted by PPV) being almost 14 times greater (72.5 per 10,000 vs 5.2 per 10,000 adults) than that recorded during MDA. Similarly for hydrocoele, after adjusting for PPV the reported prevalence was almost seven times greater (33.0 per 10,000 adults vs 4.8 per 10,000 adults) than that recorded during MDA (Tables 6 and 7).

In addition to MDA reports, further information on community-level LF morbidity prevalence was available from surveys conducted in 2006 and 2009 [24, 33]. These surveys included hydrocoele data for 26 of the 34 communities included in this study (136 [79 %] of the cases reported by SMS), and lymphoedema data for 24 communities (161 [84 %] of the cases reported by SMS, with 84 [52 %] mild, 53 [33 %] moderate and 24 [15 %] severe). In these communities, after excluding any subclinical hydrocoele cases (accounting for 52 % of cases) 268 hydrocoele cases and 286 lymphoedema cases were identified.

This discrepancy in hydrocoele cases may partially be explained by the number of hydrocoelectomies that have been undertaken in the time period between the two surveys. Anecdotal evidence provided by the doctor at Dixcove district hospital indicated that approximately one hydrocoelectomy per week is performed at the district hospital, whereas the national LF programme has performed 40 surgeries since 2009 in Ahanta West (personal communication). Therefore, whilst CHWs are unable to physically examine community members, CHW training plus community awareness of the programme appears to be a feasible approach to identifying the majority of clinical hydrocoele cases.

With regards to lymphoedema, the proportions of mild, moderate and severe cases in the alternative dataset were 42 % (120/286), 41 % (116/286) and 17 % (50/286) respectively. Given the level of disagreement between the CHWs’ and physician’s assessments of severity (Table 5), it is difficult to assess whether the CHWs under-reporting of lymphoedema in comparison to the 2006/2009 figures is affected by the severity of lymphoedema, however it’s anticipated that CHWs will have most difficulty in identifying milder cases.

### Health worker feedback

#### Malawi

The post-study questionnaire was completed by 75 % (45/60) of HSAs, with the majority of missing questionnaires being from the Kasinthula catchment area (47 % [8/17] missing). Of those that responded, 93 % (42/45) stated that it was easy to identify lymphoedema cases in their communities, whereas only 66 % (29/44, 1 missing) found hydrocoele cases easy to identify. When asked whether hydrocoele cases, once identified, were willing to have their information recorded by the health worker, 67 % (29/43, 2 missing) of HSAs responded positively.

The majority of HSAs (96 %, 43/45) stated that they did not have any difficulty submitting data by SMS. This was reflected both in the data itself where only 17 % (64/368) of SMS messages submitted contained an error, and of these 41 % (26/64) were due to the HSA entering the letter ‘o’ in place of the number ‘0’. This observation led to *MeasureSMS* being amended so to allow ‘o’ to be automatically replaced by the number ‘0’ in all numeric fields for the Ghana study. All 64 SMS messages that were sent incorrectly were later amended and resent by the HSA. The corrections were predominantly prompted by the automated error message the HSA received after submitting the incorrectly formatted SMS, although at times additional prompting was required *via* either an additional SMS or phone call.

Overall, feedback provided on the SMS system was positive, with 95 % (42/44, 1 missing) respondents identifying the ability to quickly share information as one of its main advantages, and half (22/44, 1 missing) indicating that it was easy to use. Disadvantages to the system indicated by the HSAs included cost (49 %; 21/43, 2 missing) and the unavailability of phones (51 %; 22/43, 2 missing). Suggested improvements to the system included the provision of mobile phones (39 %; 15/38, 7 missing), and increases in either phone credit or HSA allowances (26 %; 10/38, 7 missing). A further 22 % (8/38, 7 missing) also stated that they thought that community members should be followed up after the exercise in order to further assist them in managing their condition, and 16 % (6/38, 7 missing) suggested that the survey should be repeated at regular intervals. Further comments made by the HSAs can be found in Table 8.

**Table 8:** Feedback from health workers provided *via* post-study questionnaires and focus group discussions

Feedback from health workers	
“The community [has] been very happy with the programme and enjoyed it. There is a great linkage between the HSA and the community.”	*Male HSA, Malawi, aged 26-35*
“The programme is very important because these patients will be assisted accordingly; it is encouraging [the] relationship between the HSA and the community”	*Male HSA, Malawi, aged 26-35*
“It is fast information, and it is easy to get good data in our catchment area.”	*Male HSA, Malawi, aged 26-35*
“It has helped health workers to meet hydrocoele and lymphoedema cases.”	*Female HSA, Malawi, aged 26-35*
“my relationship with the community has improved due to the study and feel happy that [I] could give management advice”	*CHW, Ghana*
“I am happy that the burden of my community is now known”	Female CHW, Ghana aged 46-55
“My community will profit from this method of surveillance”	*Male CHW, Ghana aged 65+*

#### Ghana

In Ghana, 91 % (29/32) of CHWs completed the post-study questionnaire of which 79 % (23/29) stated that they easily identified lymphoedema cases whereas only 54 % (15/28) easily identified hydrocoeles. Almost all (96 %, 27/28; 1 missing) CHWs stated that, once identified, hydrocoele cases were willing to have their information recorded by the health worker.

As with Malawi, the error rate in submitted SMS messages was relatively low (16 %, 86/549), with the most common of these errors (42 %, 36/86) being that the CHW/CHW’s assistant missed out a piece of information (age, sex, condition, severity) in the SMS, with age being the most commonly missed variable (47 %, 17/36). All but two of the 86 SMS messages that were sent incorrectly were later amended and resent by the CHW, either prompted by the automated error message, or occasionally *via* either an additional SMS or phone call. The two that were not corrected were instances of where females were reported to have hydrocoeles.

As in Malawi, when asked to identify the advantages of the SMS system, 72 % (21/29) of CHWs indicated that the speed of data sharing was its greatest advantages, with 55 % (16/29) also indicating that it was easy to use. Almost one third of respondents (9/29) indicated that they could not identify any disadvantages to the system, and 21 % (6/29) indicating that charging their phone was a problem. Only 14 % (4/29) of respondents reported the availability of mobile phones as an issue. Suggested improvements to the system included providing more mobile phone credit (5/29), increasing CHW and/or community education (4/29) and increasing the CHW’s financial reward (3/29).

The focus group discussion in Ghana provided further feedback from the health workers. Seven CHWs participated in this discussion, during which methods of identifying cases, and difficulties faced differentiating between hydrocoele and hernia were discussed. With regards to identifying cases, four CHWs adopted a house to house approach whilst the remaining utilised the community announcement system to raise awareness if the survey. One individual that employed the house to house technique stated “This (hydrocoele) is a secret disease, therefore you must approach people sensitively one by one”. This statement was followed by one CHW stating that initially the men were too embarrassed to admit to having hydrocoele, but after a couple of cases came forward their reluctance reduced.

Five CHWs stated that they found differentiating between hernia and hydrocoele challenging. The two individuals that did not find this a challenge had received previous training on this condition and expressed that by taking a history they could distinguish between the two. The remaining however expressed interest in receiving further training on this topic. Interestingly, several CHWs indicated that they had felt pressured by members of their communities to report them as hydrocoele cases despite the CHW suspecting that they had (inguinal) hernias: *“The condition was in the stomach but [I] still registered because he pressured me”.*

Only one of the seven CHWs who attended the focus group had received assistance in collecting and reporting the data. When asked their opinion of this strategy, they stated that this assistance was “very very helpful”, and stated the importance of ensuring CHWs with little SMS experience had access to assistance should the exercise be repeated or extended.

As in Malawi, an important part of this method of surveillance highlighted by the CHWs was being able to “help the community through giving advice and raise awareness” whilst also reporting cases. Further CHW comments are presented in Table 8.

## Discussion

This study demonstrates the feasibility of using community-based health workers to identify LF morbidity cases, although more in-depth training is required to ensure that health workers are able to more effectively distinguish between hydrocoele and hernia. With hernia surgery training being incorporated in filarial hydrocoele surgery training programmes however, the practical importance of this distinction may decrease over time [34]. Secondly, health workers need to be trained further to ensure that milder lymphoedema cases are recognised. Previous studies conducted in Ghana [35, 36] have demonstrated the potential for health workers to diagnose lymphoedema cases using history of episodes of ADLAs in addition to a physical examination. It is crucial that early stage lymphoedema cases are identified, as these have the highest potential for halting the progression of the condition, and even reversing its effects [24]. It was noted that health workers utilised different methods to identify cases including house to house surveys and community announcement systems. Whilst each health workers is able to best judge which method will be the most effective in his or her community, due to the sensitive nature of the disease in addition to the possibility that cases are unaware of the presence or origins of their own disease, the house to house approach is preferred, and will be advocated in future applications of this approach.

Despite CHWs in Ghana having very limited experience in sending SMS messages, overall the SMS reporting system was well received and implemented by the health workers, such that almost all lymphoedema and hydrocoele cases recorded on their paper forms were successfully reported using SMS. Whilst there were minor issues such as the quality of their personal mobile phone, occasional poor network coverage and ensuring the phone was sufficiently charged, these issues were not insurmountable and had little effect on the quality of the data received. Ongoing investment in mobile network coverage in sub-Saharan Africa will increase the suitability of mHealth tools such as MeasureSMS, although continuous technical support will be required to ensure that these tools reach their full potential [37]. As the application of this reporting tool expands, with further implementation being undertaken in urban Tanzania in 2015, additional funds may become available to improve health worker mobile phone quality, additional means for charging phones such as portable battery packs and increasing mobile phone airtime.

Whilst this exercise was a standalone project designed to assess whether health workers were the appropriate target population to use to identify cases and report the cases using the *MeasureSMS* tool, once the relationship between health workers and patients has been established there is great potential to extend this exercise to include routine monitoring of patients over time thus allowing national LF programmes to more effectively monitor their progress. Therefore, the next step is to focus on delivering the appropriate care to these patients once they have been identified and reported.

## Conclusion

Given the appropriate education and tools, plus the extensive knowledge and access to members of their own community, community-based health workers are exceptionally well-placed to participate in quantifying LF morbidity burden, and other NTDs with observable symptoms. By encouraging the relationship between the health worker and the community member within an LF morbidity setting, this concept has the potential to enable national programmes to more effectively monitor their community impact in an efficient, timely and cost-effective way.

## Additional files

Additional file 1:
**Detailed information on the training sessions.**
Additional file 2:
**Pre study Questionnaire.**
Additional file 3:
**Estimated number of true cases and reported cases for the verification exercise sample size calculations.**
Additional file 4:
**Post study Questionnaire.**
Additional file 5:
**Number and location of reported cases in selected areas of Malawi and Ghana.**

## Abbreviations

CHW: Community Health Worker
GPELF: Global Programme to Eliminate Lymphatic Filariasis
HSA: Health Surveillance Assistants
KCCR: Kumasi Centre for Collaborative Research into Tropical Medicine
KNUST: Kwame Nkrumah University of Science and Technology
LF: Lymphatic Filariasis
LSTM: Liverpool School of Tropical Medicine
MDA: Mass Drug Administration
mHealth: Mobile Health
PPV: Positive predictive value
SMS: Short message service
