# Supplementary material for: Developing a community-led SMS reporting tool for the rapid assessment of lymphatic filariasis morbidity burden: case studies from Malawi and Ghana
Source: BMC Infect Dis. 2015 May 16;15:214. doi: 10.1186/s12879-015-0946-4 (PMC4455607; doi:10.1186/s12879-015-0946-4)
Supplement: Additional file 1: — Detailed information on the training sessions. [file 12879_2015_946_MOESM1_ESM.docx]

**Additional File 1: Detailed information on the training sessions**

Training sessions were conducted locally to minimise the health worker’s travel time, either at local health facilities or school buildings. Six health worker training sessions, each lasting 2-3 hours were scheduled (three per study area). At the start of each training session the health workers were informed of the purpose of the study and completed a consent form, plus a confidentiality agreement. Further, printed training materials were also distributed which included a morbidity management information booklet (Dreyer et al., 1999) (Ghana only) a data collection training booklet, a laminated summary sheet and a data entry sheet. Each document was marked with a unique health worker identification number which they used throughout the study. A period of 30 minutes to one hour was then allocated to providing information on recognising LF-related morbidity, and lymphoedema management strategies. In addition, the health workers were given information on how to assess the severity of lymphoedema. For simplicity, a three stage classification was used i.e. ‘mild’, ‘moderate’, ‘severe’ (Higher Education Academy, 2011; McPherson, Fay, Singh, Penzer, & Hay, 2006; World Health Organization, 2003). This period of training was undertaken in both the local language and English. During this period the health workers were given the opportunity to ask questions and raise any concerns they may have regarding the identification of cases.

Following this, the health workers were given further details on the data collection exercise that they were being asked to undertake. This exercise required each health worker to return to their respective catchment areas, identify all people with lymphoedema and hydrocele within the area, record the details of each identified case on a paper form (the individual’s name, village of residence, age, sex, condition and severity of condition), then send an SMS containing this information to a designated local phone. This phone number corresponded to a smartphone on which the *MeasureSMS* app was installed. The data reporting process was as follows:

- For each identified case, health workers send an SMS of the following format to a local phone number:

Case ID # Village # Health Worker ID # Sex # Age # Condition # Severity

where **Case ID** is the case number allocated to the identified individual by the health worker, **Village** is the name of the village in which the case resides, **Health Worker ID** is the individual identification number allocated to each health worker during the training session**, Sex** is M if they are male, or F if they are female, **Age** is their age in numbers, **Condition** is L if they have lymphoedema, H if they have hydrocele and B if they have both conditions, and **Severity** is 1, 2 or 3 representing mild, moderate and severe lymphoedema respectively. Note that no personal identification information is contained in the SMS.

- The SMS is received by a smartphone on which the MeasureSMS app is installed. The SMS is validated, and if it is formatted correctly, a response SMS will be sent to the health to confirm the receipt of the message. If errors are detected, a response SMS will be sent to the health worker to highlight where the mistakes have been made and to request that the message is corrected and resent.
- All SMS messages sent to the smartphone are automatically uploaded to a UK based secure cloud server once either the phone is connected to WiFi, or a 3G phone signal is detected.
- All SMS messages sent by the health worker can then be accessed in list form via a password protected web browser, with the list data including the message sent, the mobile phone number it was sent from, the date and time it was sent, and a validity code to indicate whether it was sent correctly or whether errors were made. The collated data can then be analysed and reported accordingly.

The data collection exercise training was anticipated to last 1-2 hours and was led conducted in English and the local language. During this period the health workers were given mobile phone sufficient mobile phone credit for the exercise and a practise data reporting exercise was conducted. Health workers were presented with information on fictitious LF morbidity cases and were asked to record this information on a paper form then send the corresponding SMS. At the end of the training session, health workers were asked to complete a pre-study questionnaire to obtain demographic information and assess their initial level of confidence in identifying cases in their communities, and reporting this information using the SMS system (Supplementary Material 3).
