# Supplementary material for: Developing a community-led SMS reporting tool for the rapid assessment of lymphatic filariasis morbidity burden: case studies from Malawi and Ghana
Source: BMC Infect Dis. 2015 May 16;15:214. doi: 10.1186/s12879-015-0946-4 (PMC4455607; doi:10.1186/s12879-015-0946-4)
Supplement: Additional file 2: — Pre study Questionnaire. [file 12879_2015_946_MOESM2_ESM.docx]

**Additional File 2 - Pre study Questionnaire**

The purpose of this questionnaire is to obtain information on the community that you cover, your experiences with mobile phones and your initial attitudes on the use of SMS for collecting health information in your catchment area and potential challenges you think you may face during LF morbidity data collection. All responses will remain **confidential.**

**Catchment area and demographics**

Q1. **HW ID** ……………. **Name of community**…………………………………………………………………………………………………………

Q2. **Sex**  Male □ Female □

Q3. **Age** 18-25 □ 26-35 □ 36-45 □ 46-55 □ 56-65 □ 65+ □

Q4. **Educational level** Completed primary □ Some secondary □

Completed secondary □ More than secondary □

Q5. **Literacy: Please indicate your reading and writing ability**

Reading: Unable to read □ Can read a little □ No problems with reading □

Writing: Unable to write □ Can write a little □ No problems with writing □

**LF morbidity experience**

Q6. **Excluding today, have you received any training on LF morbidity and lymphoedema management? If yes, please give the details below:** Yes □ No □ Details:………………………………………………………………………………………………………………………………………………………………………………………………………………………………………………………………………………………………………………….…………………………………

|  | | No confidence | A little confident | Quite confident | Very confident |
| --- | --- | --- | --- | --- | --- |
| Q7. | **Recognising lymphoedema and hydroceles** | □ | □ | □ | □ |
| Q8. | **Correctly assessing the severity of the lymphoedema** | □ | □ | □ | □ |
| Q9. | **Advising patients on how to manage their lymphoedema** | □ | □ | □ | □ |
| Q10. | **Advising patients on how they can access hydrocele surgery** | □ | □ | □ | □ |

**Below is a list of tasks related to LF morbidity. Please tick how confident you are in each task**

**Mobile phone experience**

Q11. **Do you have access to a mobile phone?** Yes I own one □ Yes I can borrow one □ No □

Q12. **If yes, do you use your phone to send SMS messages?** Yes □ No □

Q13. **Have you ever sent a SMS as part of your role as a HW before?** Yes □ No □

Q14. **If you answered Yes to Q13, what was the purpose of these SMS?** Please tick all that apply

| To communicate with other members of the health service e.g. other CHWs, community nurses etc. | | | 🞎 | Participated in a survey  Please specify  …………………………………………………………………. | | | | 🞎 |
| --- | --- | --- | --- | --- | --- | --- | --- | --- |
|  | | |  |  | | | |  |
| To send information relating to patients within your catchment area | | | 🞎 | To communicate with patients within your catchment area | | | | 🞎 |
|  | | |  |  | | | |  |
| Other  (Please specify)  …………………………………………………………………………….. | | | 🞎 |  | | | |  |
|  | | |  |  | | | |  |
|  | | No confidence | | | A little confident | Quite confident | Very confident | |
| Q15. | **How confident are you in writing and sending SMS messages (please tick)** | □ | | | □ | □ | □ | |

**Your opinion on SMS based health surveillance**

**Please tick below whether or not you agree with the following statements**

|  | | Yes | No | Not sure |
| --- | --- | --- | --- | --- |
| Q16. | **An SMS-based health surveillance system will result in more accurate record-keeping in comparison to a paper-based system** | □ | □ | □ |
| Q17. | **I feel comfortable with the idea of submitting patient information via SMS** | □ | □ | □ |
| Q18. | **I think that the community will benefit from an SMS-based health surveillance system** | □ | □ | □ |

Q19. **What do you think is the biggest benefit of an SMS-based surveillance system?**

…………………………………………………………………………………………………………………………………………………………………………………………………………………………………………………………………..………………………………………………………………………………………………………….................................. ............................................................................................................................................................................

Q20. **What challenges do you expect to face during data collection?**

.......................................................................…………………………………………………………………………………………………………………………………………………………….………………………………………………………………………………….……………………………………………………………………..............................................................................................................................................................................................
Q21. **How do you think the SMS-based surveillance system could be improved?**

...................................………………………………………………………………………………………………………………………………………………………..…………………………………………………………………………………………………………………………………………………………………..................................................................................................................................................................................................................................

Q22. **Do you have any other comments, either about the training received today or the study itself?**

………………………………………………………………………………………………………………………………………………………………………………………………………………………………………………………………………………………………………………………………………………………………………………………………………………………………………………………………………………………………………………………………………………………………………………………
