# Supplementary material for: Developing a community-led SMS reporting tool for the rapid assessment of lymphatic filariasis morbidity burden: case studies from Malawi and Ghana
Source: BMC Infect Dis. 2015 May 16;15:214. doi: 10.1186/s12879-015-0946-4 (PMC4455607; doi:10.1186/s12879-015-0946-4)
Supplement: Additional file 3: — Estimated number of true cases and reported cases for the verification exercise sample size calculations. [file 12879_2015_946_MOESM3_ESM.docx]

|  |  | Estimated  True Cases | Estimated Reported Cases |
| --- | --- | --- | --- |
| Malawi study area  (estimated adult pop. 50,874) | Lymphoedema | 161 | 177 |
|  | Hydrocoele | 149 | 191 |
|  |  |  |  |
| Ghana study area  (estimated adult pop. 24,835) | Lymphoedema | 78 | 86 |
|  | Hydrocoele | 73 | 94 |

**Additional File 3: Estimated number of true cases and reported cases for the verification exercise sample size calculations**. These number are based on an estimated prevalence of 31.6 lymphoedema cases per 10,000 adults and 29.3 hydrocele cases per 10,000 adults.
