# Supplementary material for: Developing a community-led SMS reporting tool for the rapid assessment of lymphatic filariasis morbidity burden: case studies from Malawi and Ghana
Source: BMC Infect Dis. 2015 May 16;15:214. doi: 10.1186/s12879-015-0946-4 (PMC4455607; doi:10.1186/s12879-015-0946-4)
Supplement: Additional file 4: — Post study Questionnaire. [file 12879_2015_946_MOESM4_ESM.docx]

**Additional File 4: Post study Questionnaire**

The purpose of this questionnaire is to obtain information of your experience of this study including any challenges you have faced, your attitudes on this method of data collection and any ways you think we may be able to improve this method of morbidity surveillance. **All responses will remain confidential.**

Please state your health worker identification number the name of your community

**HW ID …………… Community …………………………………………………….**

Your experience of lymphatic filariasis morbidity in the community

**Please tick below whether or not you agree with the following statements**

|  | | Yes (All of the time) | No (Never) | Sometimes |
| --- | --- | --- | --- | --- |
| Q1. | **It was easy to identify lymphoedema cases in my catchment area** |  |  |  |
| Q2 | **Identified lymphoedema cases were willing to participate in the survey** |  |  |  |
| Q3 | **It was easy to identify hydrocele cases in my catchement area** |  |  |  |
| Q4. | **Identified hydrocele cases were willing to participate in the survey** |  |  |  |
| Q5 | **I was confident in my ability to assess the severity of lymphoedema** |  |  |  |

Your experience of the SMS surveillance tool

**Please tick below whether or not you agree with the following statements**

|  | | Yes (All of the time) | No (Never) | Sometimes |
| --- | --- | --- | --- | --- |
| Q6. | **It was easy to submit data via SMS** |  |  |  |
| Q7 | **I was able to find all information I needed to submit data on the information sheet provided.** |  |  |  |
| Q8 | **The response SMS messages I received were easy to understand** |  |  |  |
| Q9 | **I struggled to find network coverage (signal )** |  |  |  |
| Q10 | **I deleted the SMS from my phone as soon as it was sent successfully** |  |  |  |

Q11. **How much time (in days) did you spend collecting the data?**

Less than one day 🞎 More than one day (please specify how many) 🞎

……………….. day(s)

Q12. **When did you send the SMS (tick all that apply)?**

Immediately after collecting the data 🞎 At the end of the day of data collection 🞎

Once all data had been collected 🞎 At least 1 day after I had finished data collection 🞎

**Future developments of the SMS surveillance tool**

Q13. **How important do you think LF morbidity surveillance is in your community?**

Not important at all 🞎 Slightly important 🞎 Quite important 🞎 Very important 🞎

Q14. **Would you like to be involved in future LF morbidity surveillance activities?**

Yes 🞎 No 🞎 Unsure 🞎

| Q15. **What do you think are the main benefits (if any) of using an SMS-based surveillance tool for this purpose (tick all that apply)?** | | | | | | | | | |
| --- | --- | --- | --- | --- | --- | --- | --- | --- | --- |
|  |  |  |  |  |  |  |  |  |  |
| Easy to use | | | 🞎 | Cost effective | 🞎 | Provide accurate information | 🞎 | Information can be shared quickly | 🞎 |
| Other (please specify) | | | 🞎 | ………………….…………………………………………………………………………………….…………………… | | | | | |
|  | | | | | | | | | |
| Q16. **What do you think are the main disadvantages (if any) of using an SMS-based surveillance tool for this purpose (tick all that apply)?** | | | | | | | | | |
|  |  |  |  |  |  |  |  |  |  |
|  |  | Cost | 🞎 | Time consuming | 🞎 | Not always possible to access a mobile phone | 🞎 | Difficult to use | 🞎 |
| Other (please specify) | | | 🞎 | ………………….…………………………………………………………………………………………………..……… | | | | | |

Q17. **What information/supplies do you need to assist in improving those people with lymphoedema/hydrocele in your communities to manage their conditions?**

…………………………………………………………………………………………………………………………………………………………………

…………………………………………………………………………………………………………………………………………………………………

…………………………………………………………………………………………………………………………………………………………………

Q18.**How can the SMS-based LF morbidity surveillance tool be improved?**

…………………………………………………………………………………………………………………………………………………………………

…………………………………………………………………………………………………………………………………………………………………

…………………………………………………………………………………………………………………………………………………………………

Q19: **Do you have any other comments relating to this study?**

…………………………………………………………………………………………………………………………………………………………………

…………………………………………………………………………………………………………………………………………………………………

…………………………………………………………………………………………………………………………………………………………………

Q20: Would you be willing to be considered for inclusion in a focus group discussion during which you, and approximately eight other CHWs will be asked to talk about your experiences of the SMS-based tool, and your opinions of being involved in LF morbidity surveillance in the future If you answer ‘yes’ to this question, we may contact you at the end of the study at which time we will provide you with further information on the focus group discussion. Should you be contacted, you will not be obliged to take part.

Yes 🞎 No 🞎
